# Identification and profiling of microRNAs expressed in oral buccal mucosa squamous cell carcinoma of Chinese hamster

**DOI:** 10.1038/s41598-019-52197-3

**Published:** 2019-10-30

**Authors:** Guo-qiang Xu, Li-hong Li, Jia-ning Wei, Lan-fei Xiao, Xiao-tang Wang, Wen-biao Pang, Xiao-yan Yan, Zhao-yang Chen, Guo-hua Song

**Affiliations:** 1 Laboratory Animal Center, Shanxi Key Laboratory of Experimental Animal Science and Human Disease Animal Model, Shanxi Medical University, Road Xinjian 56, Taiyuan, Shanxi 030001 China; 2 School of Public Health, Shanxi Medical University, Road Xinjian 56, Taiyuan, Shanxi 030001 China

**Keywords:** Cancer genomics, Oral cancer detection, Cancer genomics, Gene expression, miRNAs

## Abstract

MicroRNAs are known to play essential role in the gene expression regulation in cancer. In our research, next-generation sequencing technology was applied to explore the abnormal miRNA expression of oral squamous cell carcinoma (OSCC) in Chinese hamster. A total of 3 novel miRNAs (Novel-117, Novel-118, and Novel-135) and 11 known miRNAs (crg-miR-130b-3p, crg-miR-142-5p, crg-miR-21-3p, crg-miR-21-5p, crg-miR-542-3p, crg-miR-486-3p, crg-miR-499-5p, crg-miR-504, crg-miR-34c-5p, crg-miR-34b-5p and crg-miR-34c-3p) were identified. We conducted functional analysis, finding that 340 biological processes, 47 cell components, 46 molecular functions were associated with OSCC. Meanwhile the gene expression of Caspase-9, Caspase-3, Bax, and Bcl-2 were determined by qRT-PCR and the protein expression of PTEN and p-AKT by immunohistochemistry. Our research proposed further insights to the profiles of these miRNAs and provided a basis for investigating the regulatory mechanisms involved in oral cancer research.

## Introduction

As a part of head and neck squamous cell carcinoma (HNSCC), oral cancer, which is a class of common cancer in the world today, usually arises in the floor of the mouth, anterior two-thirds of the tongue, upper and lower alveolar ridges, lips and buccal mucosa, and so on^1,2^. With the changes of life style and environment, the morbidity and mortality of oral cancer become higher and higher, and the five-year survival rate is still very low, although the medical treatments such as surgery, radiotherapy and chemotherapy have been rapidly developed in recent years. For survival patients, their appearance was destroyed by the disease and in turn leading to great psychological impacts^3^. The evolution of OSCC is a perplexing regulation process of multi-gene, multi-factor, and multi-stage. In the process, many genes expression are changed and normal cell resulted in pathological process, disordered macromolecular metabolic, blocked signal transduction, and disordered in immune regulation, which leading to abnormal cell proliferation^4,5^. Thence, to provide therapeutic targets and diagnostic markers for cancer patients, it is essential to research these differentially expressed genes of oral cancer^5^.

MiRNA is non-coding and endogenous small RNA, which widely exists in eukaryotes, with a length of 20 ~ 25 nucleotides^6^. Each miRNA can regulate many target genes, which can also be controlled by multiple miRNAs^7,8^. The mature miRNA reduce stability and/or translation of target mRNA with partial or full complementary sequences, which can block the translation of the gene or cause specific disruption of the target mRNA in the complementary region, leading to the target gene silencing to participate in regulating individual growth, cell apoptosis, proliferation, differentiation and other life activities^9,10^. The recent studies have shown that the disorder in the function of miRNA leads to a variety of diseases, involving cancer, cardiovascular complications and neurological disorders, etc^11,12^. With further research, more and more researches have revealed that miRNA is widely involved in many cellular signal transduction systems and forms a complex regulatory network^13^.

Clinical treatment and prognosis are mainly based on the pathological classification of tumors, however it does not provide the information about therapeutic biology and related molecular mechanisms^14^. Therefore, it is urge to further investigate the function and application of differentially expressed miRNAs in disease diagnosis, therapeutic and prognosis. However, only a few studies are about oral cancer regulated by miRNA, and the expression feature of OSCC was rarely reported.

Chinese hamster (*Cricetulus griseus*), has a special cheek pouch tissue which is similar to human oral mucosa, so it has distinctive advantage in the research of oral diseases. Establishing animal model of oral mucosal carcinoma in Chinese hamster will help us to further understand diseases, drug screening and curative effect observation in oral, and the application of this animal model will greatly promote the research progress of oral mucosal cancer.

Based on the successful establishment of the Chinese hamster oral mucosal cancer model by coating 0.5% DMBA to both sides of cheek pouches, we firstly used high-throughput miRNA-Seq to construct the miRNAs differential expression profiles, screen differentially expressed miRNAs and verify the significant differentially expressed miRNAs by qRT-PCR, predict target genes and new miRNAs, and perform difference analysis to the GO term between Chinese hamster buccal pouch cancer and normal tissues. We also screened a landmark miRNA, and utilized it’s target genes to explore the mechanism of the PTEN/PI3K/AKT signaling pathway under the miRNA regulation during the development of oral mucosa cancer, and explored the expression level of the apoptotic genes in the downstream. These results could provide the diagnosis and treatment of OSCC in clinical with a theoretical basis.

## Result

### The effect of DMBA-induced oral carcinogenesis

**Figure 1:**
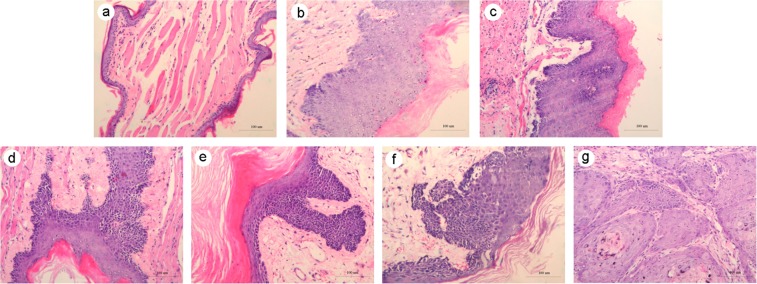
The pathological analysis of Chinese hamster buccal pouch carcinogenesis between control group and treatment group using HE (hematoxylin and eosin) stain (**a**) The control group; (**b**) The sixth week group; (**c**,**d**) The ninth week group; (**e**,**f**) The twelfth week group; (**g**) The fifteenth week group. The magnification = 200×, Scale bar = 100 μm.

To identify cancer tissues of oral mucosa cancer model of Chinese hamster, we examined the histological changes of different periods by HE stain. Compared with the control group, the treatment group had pathological changes in different periods. The Fig. 1a depicts that the normal buccal mucosa was keratinized squamous epithelium with 3–5 layers, the basal cells were arranged in an orderly manner, and the protrusion was not obvious or no protrusion. Revealed in Fig. 1b, with the passage of time, the granular layer and spinous layer of mucous epithelium became thick with mass of inflammatory cells infiltrated under the mucous membrane. The proliferation of basal cells in complex layer was obvious, the mutation became wide showing the shape of papillate and nail, a few epithelial cells were atrophied and the lamina propria showed an obvious inflammatory reaction, which was the pathological manifestation of ulcerative tissue in Fig. 1c,d. Further, the morphology and volume of epithelial cells are varied, the nucleus were irregular, and the abnormal proliferative cells had reached more than 2/3 in the epithelial cell layer in Fig. 1e,f. When it reached the 15th week, the epithelial cells and nuclei showed obvious polymorphisms. The cells broke through the basement membrane, infiltrating the lamina propria and connective tissue, and many tumor islands emerged. The tumor cells have atypical mitosis, accompanied with early keratinization, nucleolus enlargement, and highly differentiated squamous cell and invasive cancer (Fig. 1g). According to the WHO criteria^15^ for cancer diagnosis, it is known that the treatment group has been in the state of squamous cell carcinoma at 15th week. Therefore, oral squamous cell carcinoma animal model was established successfully.

### Sequencing and analyzing of small RNAs from cancer and normal samples

Small RNA libraries from cancer and normal group were sequenced using Illumina® technology. The clean reads were in the range of 15–35 nt, among which the most clean sequences were 22 nt long in all libraries. However, the majority of unique clean reads were in the range of 20–25 nt. Match the length of clean reads with the genomic reference sequence, perfect match rate of all samples were more than 60%.

To identify miRNAs in Chinese hamster, all perfectly matched sRNA sequences were compared to known miRNA database. The amount of Total Clean Reads Mature in the group of Cancer 1, Cancer 2, Cancer 3, Normal 1, Normal 2, and Normal 3, was 4301349, 5771545, 9367231, 7450887, 7678749 and 7431537 respectively, after further classification of the data, 3249, 3041, 3984, 3795, 3681 and 3634 Unique Clean Reads Mature for the different groups were obtained separately. In the process of precursor convert to mature body of miRNA, the Dicer enzyme, which made mature body of miRNAs have a certain base preference in the specific shear, was required. Through statistical base preference, the structure of miRNAs were clarified. The base distribution of known miRNAs in the normal and cancer groups shows that the first site of miRNA is U, followed by A. Furthermore, the non-coding RNAs excepting known miRNAs were grouped into several categories, including rRNAs, snRNAs, snoRNAs, tRNAs and other small RNAs (Undef). There were 2377095, 2122062, 281519, 1170988, 338026 and 251156 Total Clean Reads Novel, and 561, 559, 834, 633, 607 and 585 Unique Clean Reads Novel in the 6 groups that were sequenced. Similarly, the base distribution of novel miRNAs in cancer and normal tissues was statistically analyzed, and the first site of miRNA was found to be U. In addition, it was shown that A was higher in site 15 and C was higher in site 17.

### Differential expression analysis of known and novel miRNAs in the cancer and normal samples

**Figure 2:**
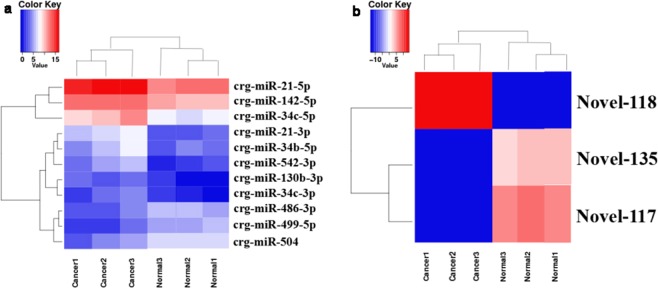
Cluster analysis of 11 known (**a**) and 3 novel (**b**) significantly differentially expressed miRNAs in each simple. In the figure, the expression change of miRNAs is indicated by the change of color. Blue indicates low expression level, red indicates high expression level.

To investigate the expression pattern of miRNA in buccal pouch squamous carcinoma tissues of Chinese hamster, we compared the normalized expression values of miRNAs between normal and cancer groups. According to the criteria described in “Methods”, 268 miRNAs were determined as the differentially expressed known miRNAs, 137 miRNAs were up-regulated and the other miRNAs were down-regulated. From the 268 differentially expressed known miRNAs we screened 11 miRNAs which log_2_|(FoldChange)| > 2 and listed them in Table 1. For novel miRNAs, there were 208 novel miRNAs differentially expressed between normal and cancer groups (112 novel miRNAs up-regulated and 96 novel miRNAs down-regulated), including 3 significantly differentially expressed novel miRNAs with *p*-value < 0.05 (1 miRNAs up-regulated and 2 miRNAs down-regulated) (Table 2). Then cluster analysis was performed on the log_2_ (RPM) values of the significantly differentially expressed miRNAs in each sample (Fig. 2). It directly reflected the degree of similarity between different samples. We could get the clustering coefficient between each sample and gene, and the clustering results of the whole samples were obtained finally (Fig. 2).

**Table 1:** Significantly differentially expressed known miRNAs.

miRNA Name	Sequence (5′-3′)	Normalized Expression Level		log_2_FoldChange	p	Type	counts of target gene
		Cancer	Normal				
cgr-miR-542-3p	UGUGACAGAUUGAUAACUGAAAG	349.1586376	37.22293902	3.229618787	0.00348059	up	205
cgr-miR-130b-3p	CAGUGCAAUGAUGAAAGGGCAU	109.5301061	19.077294	2.521399018	0.025768873	up	152
cgr-miR-142-5p	CCCAUAAAGUAGAAAGCACUAC	96094.21987	17489.09373	2.457994122	0.0109149	up	55
cgr-miR-34c-3p	AAUCACUAACCACACGGCCAGG	100.1299789	16.37065933	2.612689648	0.029082558	up	64
cgr-miR-34c-5p	AGGCAGUGUAGUUAGCUGAUUGC	17968.01233	1656.979007	3.438803593	1.19E-05	up	656
cgr-miR-34b-5p	AGGCAGUGUAAUUAGCUGAUUGU	771.2881636	100.3426342	2.942335253	0.046276965	up	372
cgr-miR-21-3p	CAACAGCAGUCGAUGGGCUGUC	1384.549882	81.59257549	4.084835341	8.06E-06	up	626
cgr-miR-21-5p	UAGCUUAUCAGACUGAUGUUGA	892190.6025	68507.88498	3.703010001	3.64E-06	up	44
cgr-miR-504	AGACCCUGGUCUGCACCUCUAUC	192.4795887	1246.106273	-2.694649742	0.015868948	down	799
cgr-miR-499-5p	UUAAGACUUGCAGUGAUGUUUA	51.58103734	543.2782934	-3.396778713	0.000709348	down	46
cgr-miR-486-3p	CGGGGCAGCUCAGUACAAGACG	82.12365246	579.6180494	-2.819232823	0.010386505	down	467

**Table 2:** Significantly differentially expressed novel miRNAs.

miRNA Name	Sequence (5′-3′)	Normalized Expression Level		p	Type	counts of target gene
		Cancer	Normal			
Novel_117	GUUUAUGUAUGUGUAUAUGUAU	0	1911.022867	0.046184485	down	10
Novel_118	UAACACUGUCUGGUAACGAUGU	161874.5039	0	9.63E-06	up	180
Novel_135	UAGGCUAGAAAGAGGCUGGGGAU	0	176.4588548	0.046184485	down	287

### Verification of miRNAs through qRT-PCR

**Figure 3:**
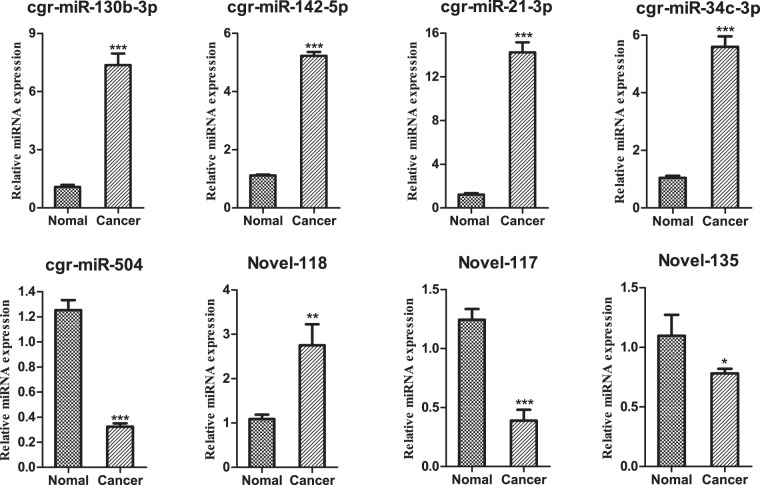
qRT-PCR analysis expression levels of miRNAs in the normal and cancer group. 5 s rRNA was used as a reference. *P < 0.05, **P < 0.01 and ***P < 0.001.

We performed qRT-PCR analysis on the 5 significantly differentially expressed known miRNAs and 3 significantly differentially expressed novel miRNAs. Compared with the normal group, crg-miR-130-3p (7.372 ± 0.3416 vs 1.085 ± 0.06151), crg-miR-142-5p (5.228 ± 0.07653 vs 1.117 ± 0.01856), crg-miR-21-3p (14.250 ± 0.5306 vs 1.214 ± 0.08204), and crg-miR-34c-3p (5.598 ± 0.2093 vs 1.043 ± 0.04295) increased extremely significant (*p* < 0.001) and had relatively high and stable expression levels in the cancer group. Similarly, Novel-118 (2.752 ± 0.2754 vs 1.088 ± 0.05883) was highly expressed (*p* < 0.01) too. However, the expression of crg-miR-504 (0.3235 ± 0.01582 vs 1.254 ± 0.04585) and Novel-117 (0.3891 ± 0.05357 vs 1.2450 ± 0.05247) were significant lower than the normal group (*p* < 0.001), and Novel-135 (0.7811 ± 0.02293 vs 1.097 ± 0.1015) was also lower (*p* < 0.05). The qRT-PCR results indicated that the expression level of the miRNAs was consistent with the results of Illumina sequencing (Fig. 3).

### Target prediction and functional analysis of significantly differentially expressed miRNAs

To better understand the functions of miRNAs, MiRanda (http://www.microrna.org/)^16^ was used to predict the target genes of the 11 known and 3 novel significantly differentially expressed miRNAs. The results showed that there were several or even hundreds of target genes in each miRNA (Tables 1, 2).

**Figure 4:**
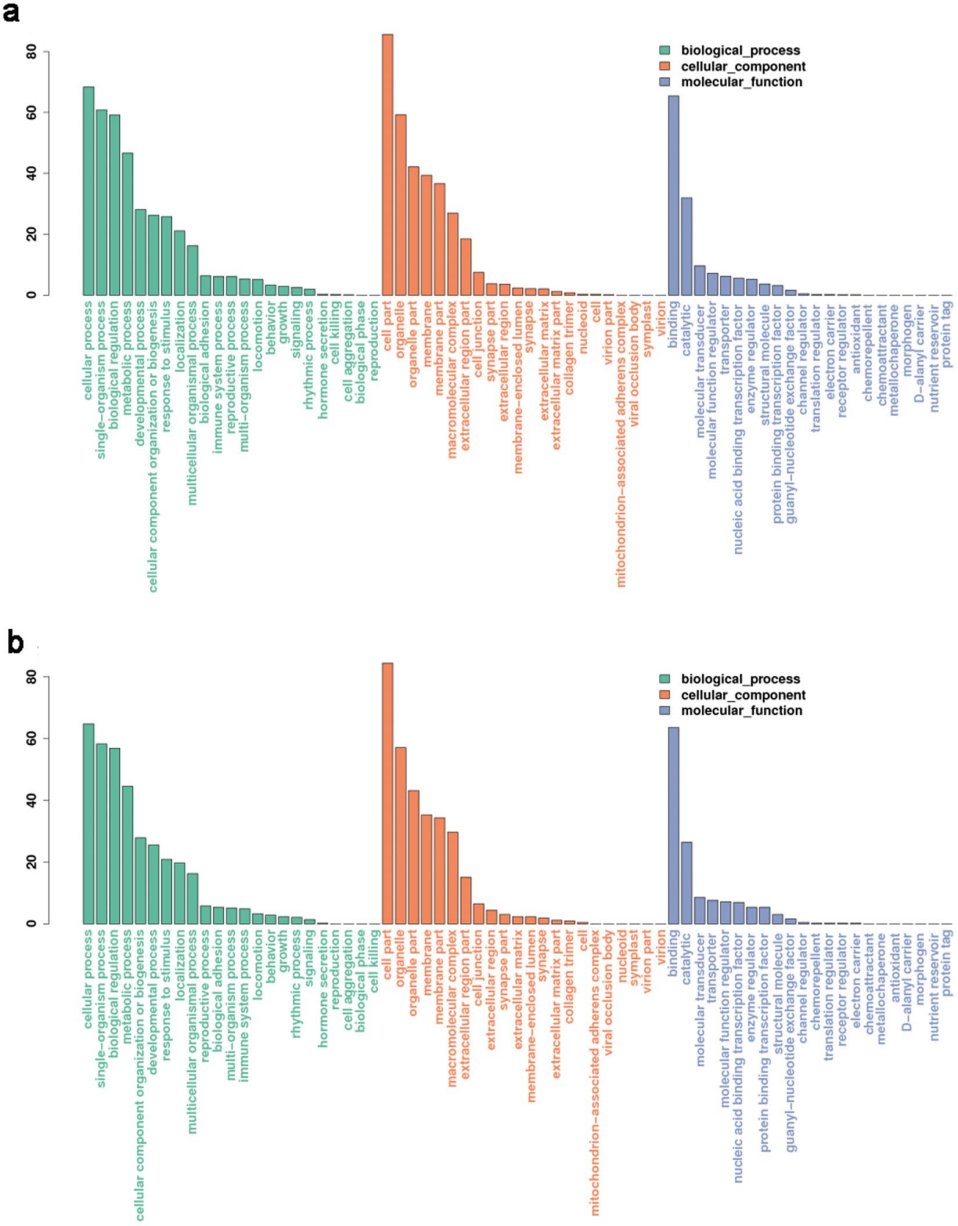
GO statistical histogram of the target genes of significantly differentially expressed miRNAs. (**a**) The GO statistical histogram of the target genes of the known miRNAs. (**b**) The GO statistical histogram of the target genes of the novel miRNAs. The X axis is the type of Ontology. The green represents the biological process, and the orange represents cell component, while the blue indicates the molecular function. The Y axis is the proportion of target genes annotated to the Class in all annotated target genes. Since the same gene appears repeatedly in different Class, the percentage of all columns is more than 100 percent.

**Figure 5:**
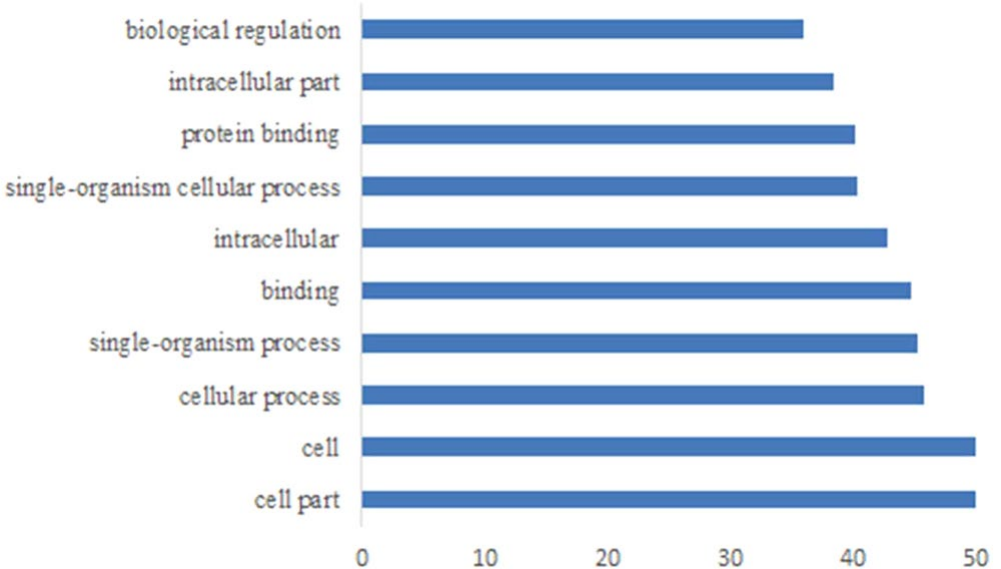
The top ten GO statistical histogram of the target genes of miR-21. X axis is the value of −log (Corrected P-Value).

To describe the associated biological processes, cellular components, and molecular functions of miRNA target gene products, we conducted gene ontology (GO) analysis (Fig. 4). Target genes of both known (Fig. 4a) and novel (Fig. 4b) mainly enriched in cellular processes, single-organism process and biological regulation, which account >80% changes of biological process. In the cellular components, the target genes were largely responsible for cell part, organelle and organelle part. Furthermore, for known miRNAs, we obtained a number of GO entries after enriched screening. The *q* < 0.05 was a threshold, 340 biological processes, 47 cell components, and 46 molecular functions were projected. However, for novel miRNAs, there was only 1 GO entry. To learn more about the functions of the interesting miRNA, we conducted GO analysis of the target genes of miR-21(Fig. 5).

### Expression of PTEN and p-AKT

**Figure 6:**
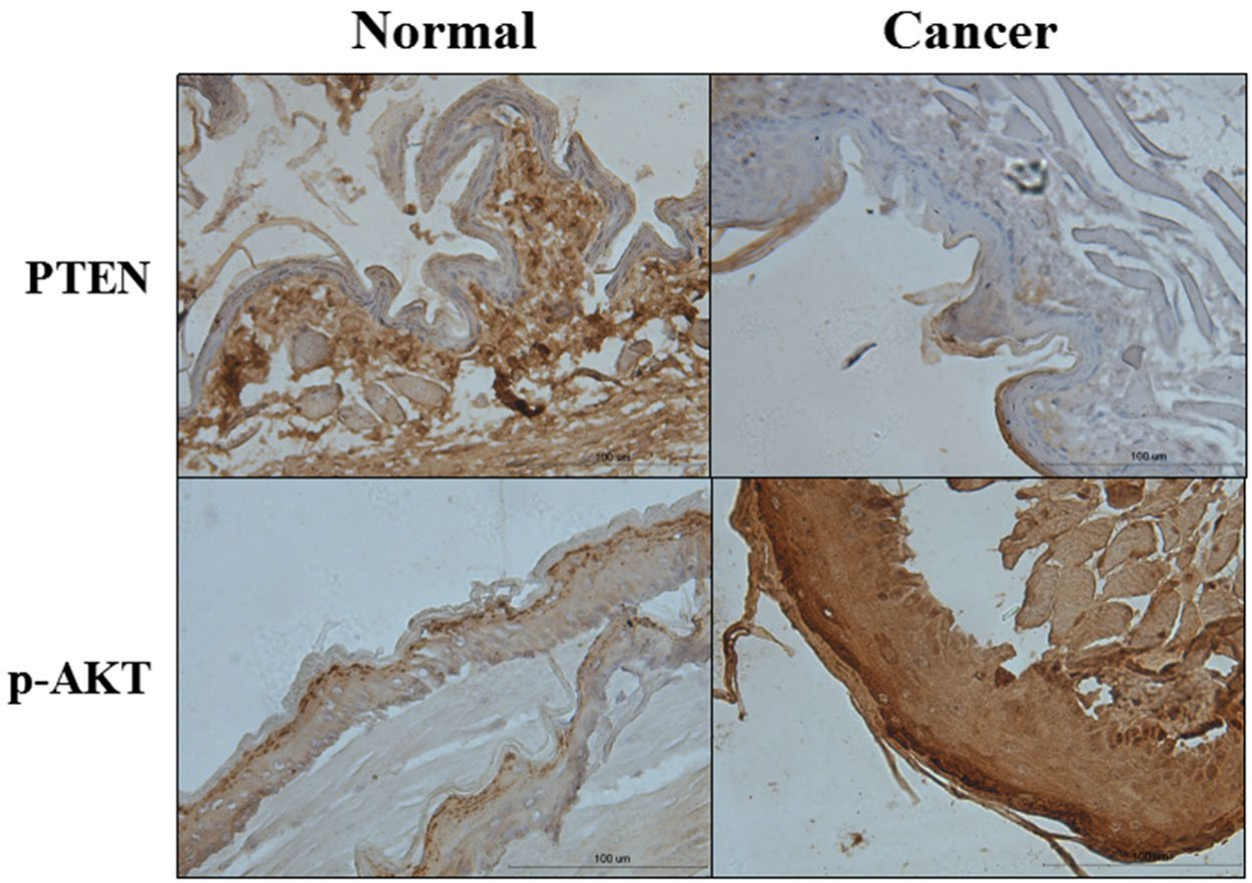
PTEN and p-AKT protein expression of pouch in the normal and cancer group by Immunohistochemistry (IHC × 200, Scale bar = 100 μm).

The miRNA targets prediction software and a lot of related studies demonstrate that PTEN is one of the most common target genes of miR-21. The mutation or deletion of PTEN causes the continuous activation of AKT, which enhances the transcriptional and expressive activity of the anti-apoptotic gene. The immunohistochemistry was used to test expression of the protein of PTEN and p-AKT. The PTEN was significantly reduced (*p* < 0.001), however, the p-AKT was significantly risen (*p* < 0.001) in cancer groups (Fig. 6).

### Expression of apoptotic gene

**Figure 7:**
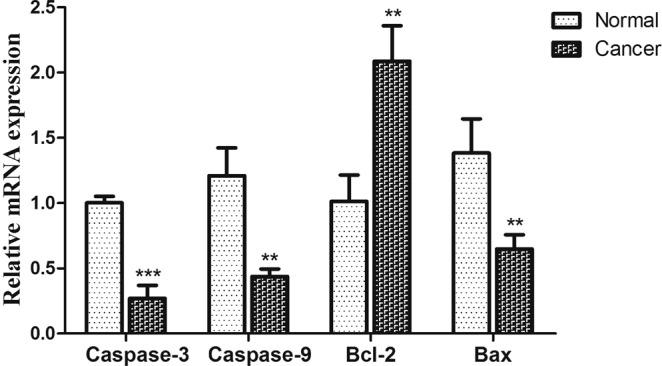
Expression of Caspase-3, Caspase-9, Bcl-2 and Bax in normal and cancer group. β-Actin was used as a reference. The values are expressed mean ± SE (n = 3).*P  < 0.05;**P  < 0.01, and ***P *<* 0.001.

**Figure 8:**
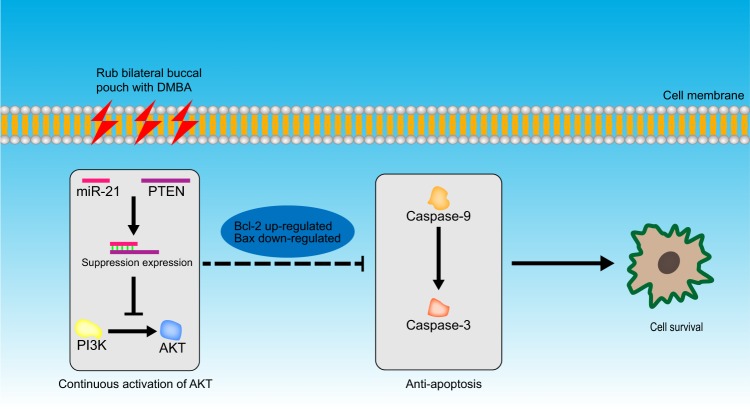
the Regulatory mechanism.

PTEN and p-AKT are associated with cell apoptosis, and play a crucial role in tumor progression, so the mRNA level of the Caspase-9, Caspase-3, Bcl-2 and Bax, which are considered as the dominant role during apoptosis, were detected by qRT-PCR. The Fig. 7 depicts that the expression of Caspase-3 was extremely decreased (P < 0.001), Caspase-9 and Bax were down-regulated significantly (P < 0.01), while the Bcl-2 was rose in squamous cell carcinoma tissues, with statistically significant difference (P < 0.01). The regulatory mechanism was depicted in the Fig. 8.

## Discussion

The oral cancer animal model was established in 1954 by Sally on the oral buccal pouch mucosa of golden hamsters. From then on, the model has become one of the typical animal models of oral carcinoma^17^. Chinese hamster and golden hamster belong to different species, but both have cheek pouches. Chinese hamster has a strong vitality and small body (about 9 cm long) which makes it easy to operate by a single hand, thence, it has distinctive advantage in the research of oral diseases^18^. In current research, we constructed the oral cancer animal model on the buccal mucosa of Chinese hamsters successfully by using 5% DMBA acetone solution. High-throughput sequencing on Chinese hamster oral squamous cell carcinoma model could profiles thousands of expression patterns of miRNAs simultaneously, predicts their target genes and potential functional network(which was consist of some differentially expressed signaling pathways in oral mucosal cancer tissue and are likely responsible for the occurrence and evolution of oral carcinoma)^19^.

MiRNA is a double-edged sword in cancer, it could be included as a proto oncogene in the occurrence and evolution of malignant tumor, but on the other hand it also acts as a tumor suppressor to inhibit the carcinoma formation^20,21^. Researches by Wei Jiang *et al*. manifested that miRNAs can interact with some small molecules to affect the cancer development^9,10,22^. A mushrooming number of evidence has proved the importance of miRNAs in cancers, indicating their possible application as diagnostic, prognostic and predictive biomarkers. In this study, we constructed Chinese hamster buccal pouch carcinoma model and built small RNA libraries which will benefit clinical research and treatment of oral cancer.

We predicted that miRNA could influence the formation of OSCC by regulating multiple target genes. In order to find the function of their target genes, we analyzed target genes by GO. Functional analysis of miRNA target genes revealed that miRNAs primarily regulated the activity of nucleotide binding enzymes, organelle parts and cell components, which might affect biological regulation. Thus, it could be speculated that differentially expressed miRNAs might affect OSCC development via regulating target genes to adjust the activity of nucleotide binding enzymes, organelle parts and cell components.

GO enrichment analysis was applied to predicted target genes of all miRNAs, and 340 biological processes, 47 cell components, and 46 molecular functions was obtained. However, there are a few significantly different novel miRNAs, and a few targets for novel miRNAs. Meanwhile, we have used targets of novel miRNAs for functional analysis, but the corresponding functions of the novel miRNAs are less.

The GO terms that target genes mainly enriched in were cellular processes, single-organism process and biological regulation which were belong to biological process, and cell part, organelle and organelle part which were belong to cellular components. MicroRNA can affect the growth, necrosis and apoptosis by acting on massive biological processes of OSCC. JS Kim^23^, *et al*. found that miR-203 induces apoptosis in the YD-38 cell line by inhibiting bmi-1 expression. The experimental research of Min, Seung-Ki, *et al*.^24^ revealed that the expression of exogenous miR-146a-5p could activate the downstream JNK of its target, further has an impact on apoptosis and proliferation of cells with OSCC. An interesting result was depicted by Chou, S.-T *et al*. *in vitro* experiment, miR-486-3p inhibits growth and promotes apoptosis by targeting DDR1, which is consistent with the effect of knockdown DDR1^25^. Meanwhile, miRNAs can also affect the occurrence and development of OSCC by regulating the specific cellular components in OSCC cells. Chen YH *et al*.^26^ found that miRNA-10a could cause GLUT1 high expression, which leads to the acceleration of glucose metabolism, and further promotes the growth of OSCC cells. Xu YX, *et al*.^27^ validated the upregulation of miR-4513 downregulates the CXC ligand 17 (CXCL17) expression, then promotes cell proliferation, migration, invasion, and, at the same time, inhibits apoptosis.

An ocean of researches depicted that miR-21 is widely high expression in many malignant tumors and plays a crucial regulatory role in their growth and apoptosis^28^. In the early study, Soga D *et al*. investigated the expression profiles of 29 OSCC tumor samples which included various stages and histological grades, finding that miR-21 *et al*. were up-regulated, while miR-133a, miR-376c, and miR-411, *et al*., were down-regulated in OSCC^29^. Xiaoyi Wang *et al*. applied 5% DMBA to hamsters three times a week to construct the animal model of OSCC, and then used microRNA microarray to analyze the gene expression profile of model group. Finally they found that there were 5 significantly up-regulated microRNAs (miR-21, *et al*.) and 12 down-regulated microRNAs in oral cancer groups^30^. Generally, not only in OSCC, miR-21 is also highly expressed in lung cancer, cervical carcinoma, B cell lymphoma, liver cancer, multiple myelomas, pancreatic cancer, leukemia and so on^31^. MiR-21 could be used not only as a biomarker, but also a therapeutic target. Related experiments by Li Xu, Y *et al*. demonstrated that knockdown of miR-21 could significantly inhibit the migration of breast cancer cells *in vitro* and the growth of transplanted tumors *in vivo*^32^. In our current study, both cgr-miR-21-3p and cgr-miR-21-5p were increased in OSCC, which consistent with previous studies, meanwhile, stressed that miR-21 has a pivotal role on oral cancer.

According to the previous report and our prediction, plenty of tumor suppressor genes including PDCD4 and PTEN were confirmed as targets of miR-21^33^. In addition, Zheng, Y *et al*. reported that miR-21 acts as a regulator factor in many signaling pathways such as Wnt/β-catenin and PI3K/Akt^34^. Meanwhile, PTEN is a direct target in downstream of the PI3K/Akt pathway and is associated with apoptosis and tumor growth^35^. The mutation or deletion of PTEN causes the continuous activation of AKT, which enhances the transcriptional and expressive activity of Bcl-2, at the same time Serl84 residue of Bax was deactivated, thereby suppressing apoptosis and promoting cell survival^36^. The number of Bcl-2/Bax increases correspondingly, which can inhibit cytochrome C release in mitochondria^37^. When cytochrome C release is inhibited, it cannot combine with Apaf-1 (apoptotic factor 1) under dATP condition to inhibit Caspase-9 and further inhibits Caspase-3(central effector caspase)^38^, which leads to close the copy and repair program of the DNA, block the splicing of the RNA, further degrade the DNA. Then it leads to nuclear breakdown, which induces the cell to send signal to the outside so that it can be surrounded. At the last, the phagocytic cells were disintegrated and wrapped up to form an apoptotic body that eventually formed apoptosis^39^.

In our research, the PTEN was significantly reduced, while p-AKT was increased in cancer group. Meanwhile, in squamous cell carcinoma tissue, Bcl-2 expression was higher, while the Caspase-3, Caspase-9 and Bax were significantly decreased. These results supported the opinion that the miR-21 may affect the occurrence of oral cancer by restraining the expression of PTEN, which regulated the expression of apoptotic protein through the PI3K/Akt signal pathway. In clinical, the expression characteristics of these proteins can guide the diagnosis of OSCC. These studies may also guide the study of clinical drugs, especially the research of targeted drugs for these significantly differentially expressed miRNAs.

Altogether, Chinese hamster could be, as a fantastic animal model for oral cancer research, to identify the miRNA profiles in OSCC. The results demonstrated that miR-21 regulated apoptotic protein expression through the PI3K/Akt signal pathway. In order to better guide the clinic, we will do functional and mechanistic research of the miR-504 and miR-34c in oral cancer. From the perspective of comparative medicine, we would provide theoretical basis and scientific evidence for the research that miRNA leads to the disorder of molecular mechanism in the evolution of OSCC.

## Materials and Methods

### Animals

Chinese hamsters (n = 60, male, 8-10 weeks old, 22 +/− 2 g) were provided by the Laboratory Animal Center of Shanxi Medical University (Taiyuan, China). The production license number is SCXK [Jin] 2015-0001. All the animals were randomly divided into three groups as follows: the control group (n = 24), the solvent control group (n = 12), and the treatment group (n = 24) . Our study was approved by the Institutional Animal Care and Use Committee of Shanxi Medical University (IACUC 2017-016). Animal experiments were carried out strictly in accordance with the operating rules formulated by the IACUC and accepted the supervision and inspection. The animals were raised in a barrier environment (25 °C, 45% humidity, 12:12 light: dark cycles) where water and food are freely available (SYXK [Jin] 2015-0001). Those normal samples have rich blood vessels, with dense microvasculature. As a result of these organizations is light color, good pervious to light, they can be prepared for cheek pouch small room. In addition, the control group had good sensitivity, spirit and appetite, and the coat color was bright and smooth. The treatment group/solvent control group were rubbed bilateral buccal pouch with DMBA/acetone solution by a cotton swab three times a week for 15 weeks, and fasting 2 h after using rubber suction bulb to dry. The control group received no treatment.

All buccal pouch samples of Chinese hamsters were collected under pentobarbital anesthesia for histopathological examination, high-throughput miRNA-Seq, and qPCR validation, on the week 15. All samples from three groups were immediately isolated and randomly divided into two sections: The one was fixed in 4% paraformaldehyde to histopathological detection, and the other was frozen in liquid nitrogen and stored at −80 °C for RNA sequencing and gene expression research.

### Histopathological analysis

Pouch tissue samples were fixed in 4% paraformaldehyde for about 24 hours, transferred to 70% ethanol, and then processed in a graded series of ethanol solutions. The samples were subsequently embedded in paraffin, serially sectioned at 4 μm and stained with hematoxylin and eosin (HE) for histopathological examination. The pouch pathological changes were determined according to the 12 grade record in the WHO standard^15^.

### RNA extraction and quality control

According to the pathological results, we found that the squamous cell carcinoma model was successfully constructed at the 15 week. So we selected the cancer group and the normal group at the 15 week for RNA sequencing. Six proper amount of tissue samples were used to construct small RNA libraries in this study. The 6 samples, including normal (n = 3) and cancer group (n = 3), were individually subjected to total RNA extraction using the TRIzol reagent (Invitrogen, Carlsbad, CA, USA) according to the manufacturer’s instructions. After diluting RNA (>10ug) according to certain proportion, 1% agarose gel electrophoresis was used to detect the degradation of RNA samples and the presence of impurities. Then the purity of samples was detected by using Kaiao K5500 spectrophotometer (Kaiao, Beijing, China). The concentration, 28S/18S and RIN of the extracted total RNA was detected using an Agilent 2100 RNA Nano 6000 Assay Kit (Agilent Technologies, CA, USA). The RIN was about 7.0 and A_260_/A_280_ was >1.8–2.0 for all samples. Qualified RNA samples were used for library construction and deep sequencing.

### Small RNA library construction, sequencing and analysis

After the total RNA sample test, a total of 15–35 nt RNA fragments were excised, purified from a PAGE gel, and ligated with 5′ and 3′ adaptors using T4 RNA ligase. Reverse transcription followed by PCR was used to create cDNA based on the small RNA ligated with 3′ and 5′ adapters. Subsequently, the amplified cDNA constructs were purified from agarose gel, in preparation for sequencing analysis using the Illumina HiSeq 2500 Analyzer (Illumina, CA, USA) according to the manufacturer’s instructions.

### Identification of known and novel miRNAs

Initially, the raw sequences were processed by Illumina’s Genome Analyzer Pipeline software (Annuogene, Beijing, China). Then to get clean reads, the adapter sequences, low quality sequences and low-copy sequences were removed. After the basic analysis, the qualified sequences were mapped onto the Cricetulus griseus reference sequence using the genome alignment analysis software Bowtie2 (http://computing.bio.cam.ac.uk/local/doc/bowtie2.html)^40^ and the length distribution of them was calculated; the known miRNA sequences were detected according to miRBase 21.0 (http://www.mirbase.org/)^41^; rRNA, tRNA, snRNA, and snoRNA were identified against Rfam (11.0) (http://rfam.xfam.org/)^42^ and NCBI GenBank database. The reads which cannot be matched to any of the above databases were marked as ‘Undef’. To identify novel miRNAs, rest of the unmapped small RNA sequences were searched by software miRDeep2 (http://biowulf.nih.gov/apps/mirdeep2.html)^43^. The mappable sequences were then folded into a secondary structure using RNAfold software with default parameters. Only the non-coding sequences could form a perfect stem-loop structure and meet the criteria for miRNAs prediction were then considered to be a novel miRNA candidate.

### Differential expression analysis

Differential expression analysis was performed between cancer group and normal group according to DESeq software (1.160). In the first step, the clean reads of each miRNA were normalized [the number of reads per million (RPM) normalized expression = number of reads mapping to miRNA * 1,000,000/number of reads in clean data]. The statistics was performed by DESeq software, and the adjusted *p*-value less than 0.05 and log_2_|(FoldChange)| more than 1 were considered as significant differences. Furthermore, clustering analysis was performed for the DE-miRNAs between normal and cancer tissues using a hierarchical clustering method. According to the expression of miRNA in each sample, the Euclidean distance is calculated after taking the logarithm of 2 as the base. Finally, the systematic clustering method was used to obtain the clustering results of the significantly differentially expressed miRNA between the samples.

### Target gene prediction of miRNAs and functional analysis

Target genes of miRNAs were predicted using MiRanda (http://www.microrna.org/)^16^. Numerous target sequences were assigned to various non-redundant (Nr) proteins, Uniprot, GO, COG. The biological processes, molecular functions, and cellular components of the target genes were examined using the agriGO online tool and according to the GO terms in the database (http://www.geneontology.org/) to perform Gene Ontology (GO) annotation and enrichment analysis. The statistical test method was set as Hypergeometric. The formula of *p* value was:

$${\rm{P}}=1-\mathop{\sum }\limits_{i=0}^{{\rm{m}}-1}\frac{(\begin{array}{c}M\\ {\rm{i}}\end{array})(\begin{array}{c}N-M\\ {\rm{n}}-{\rm{i}}\end{array})}{(\begin{array}{c}N\\ {\rm{n}}\end{array})},$$

N is the number of all genes with GO annotation, n is the number of target gene candidates in N, M is the number of all genes annotated to a certain GO term, and m is the number of target gene candidates in M. We used the Bonferroni correction to obtain a corrected *p* value. GO terms with corrected *p*-value ≤ 0.05 were defined as significantly enriched in the target gene candidates. The formula of the false discovery rate (FDR) was the same as the *p* value in GO analysis. Genes with FDR ≤ 0.01 were considered significantly enriched target gene candidates.

### Quantitative Real-time PCR analysis

Total RNA was extracted with TriPure Isolation Reagent (Roche, Switzerland). Complementary DNA (cDNA) was synthesized with All-in-One miRNA First-Strand cDNA Synthesis Kit (GeneCopoeia, USA) in a total reaction volume of 25 uL. The primers used for amplification were obtained commercially from GeneCopoeia (Guangzhou, China) (Table 3). Quantitative Real-time PCR was performed on StepOne Plus (ABI, USA), using All-in-OneTM miRNA qRT–PCR Detection Kit and following the manufacturer’s protocol (GeneCopoeia, USA). 5 s rRNA was universal adaptor primer which was used for normalizing the expression of miRNA. There were 3 subjects used for the qRT-PCR analysis, and they were separated from the ones used for the RNAseq analysis.

**Table 3:** The primer sequences of miRNA. 5 s rRNA was universal adaptor primer which was used for normalizing the expression of miRNA.

miRNA	Sequence	Amplification fragment size	Annealing temperature (°C)
cgr-miR-130b-3p	AGTGCAATGATGAAAGGGCAT	75	60
cgr-miR-142-5p	GCCCATAAAGTAGAAAGCACTACAA	77	60
cgr-miR-34c-3p	AATCACTAACCACACGGCCA	74	60
cgr-miR-21-3p	CAACAGCAGTCGATGGGCT	73	60
cgr-miR-504	AGACCCTGGTCTGCACCTCTA	75	60
Novel_118	GCTAACACTGTCTGGTAACGATGTA	78	60
Novel_117	CCCGGTTTATGTATGTGTATATGTATAAA	80	60
Novel_135	GGCTAGAAAGAGGCTGGGGAT	75	60
5s rRNA	/	72	60

The primers of Bax, Bcl-2, Caspase-3 and Caspase-9 were designed and synthesized by BGI tech (Table 4). qRT-PCR was performed on StepOne Plus (ABI, USA), using PrimeScript^TM^ RT Master Mix (Perfect Real Time) and SYBR® Premix Ex Taq^TM^ II (TaKaRa, Japan). Thermal cycling conditions are in accordance with the product manual.

**Table 4:** The primer sequence of apoptotic genes used for qRT-PCR.

Gene	Sequence	Annealing temperature (°C)
Bax	F: 5′-CTCAAGGCCCTGTGCACTAAA-3′ R: 5′-CCCGGAGGAAGTCCAGTGT-3′	60
Bcl-2	F: 5′-GGAGGCTGGGATGCCTTTG-3′ R: 5′-GTGAGCAGCGTCTTCAGAGACA-3′	60
Caspase-3	F: 5′-AGGCCGACTTCCTGTATGCTT-3′ R: 5′-TGACCCGTCCCTTGAATTTC-3′	60
Caspase-9	F: 5′-GAGAGACATGCAGATATGGCATACA-3′ R: 5′-CAGAAGTTCACGTTGTTGATGATG-3′	60
β-Actin	F: 5′-CTGAGCCAGATGCTGTCCCATA-3′ R: 5′-GACACCATCCAAGGTCTCGATGTA-3′	60

### Immunohistochemistry analysis

The SABC two-step method for immunohistochemical staining was used to determine the expression of PTEN, p-Akt proteins in OSCC tissues. Each tissue paraffin block was cut into serial 4 μm sections, afterwards, dewaxed and hydrated with gradient alcohol. Endogenous peroxidase activity was blocked by incubation with 3% hydrogen peroxide (H_2_O_2_). Heat induced epitope retrieval (HIER) in a microwave was performed with citrate buffer pH 6.0. Moreover, the sections were incubated with the first antibody at 4 °C overnight, the antibody concentration of PTEN diluted 1:100, while the first antibody concentration of p-Akt diluted 1:50. Furthermore, they were incubated with horseradish peroxidase-labeled goat anti-mouse secondary antibody for 30 min at 37 °C followed by SABC for 30 min at 37 °C. In addition, slides were incubated for 5–30 min in DAB (3, 3-diaminobenzidine, Biogenex) followed by counterstaining with hematoxylin. Last, each sample was hydrated with gradient alcohol and sealed. All the reagents were from Wuhan Boster Biological Technology Ltd., Wuhan, China. The cells with visible yellow or brown cytoplasm or cell membrane were identified as positive. Semi-quantitative analysis was performed by Image Pro Plus (IPP) analysis software. Areas positive for a particular color of dye were selected and software was used to calculate the optical density. There were 5 subjects used for immunohistochemistry analysis of AKT and PTEN protein.

### Statistical analysis

All qRT-PCR and immunohistochemical experiments were performed in triplicate. Data are presented as means ± SE. Statistical analysis was performed using SPSS (version 16.0). *P* < 0.05 was considered statistically significant by Student’s t-test for two groups. Correlation was analyzed with two-tailed Spearman^’^s correlation analysis.

## Supplementary information

Dataset 1
